# Allogeneic Transplantation of Periodontal Ligament-Derived Multipotent Mesenchymal Stromal Cell Sheets in Canine Critical-Size Supra-Alveolar Periodontal Defect Model

**DOI:** 10.1089/biores.2015.0043

**Published:** 2016-01-01

**Authors:** Yuka Tsumanuma, Takanori Iwata, Atsuhiro Kinoshita, Kaoru Washio, Toshiyuki Yoshida, Azusa Yamada, Ryo Takagi, Masayuki Yamato, Teruo Okano, Yuichi Izumi

**Affiliations:** ^1^ Department of Periodontology, Graduate School of Medical and Dental Sciences, Tokyo Medical and Dental University, Tokyo, Japan.; ^2^ Institute of Advanced Biomedical Engineering and Science, Tokyo Women's Medical University, Tokyo, Japan.; ^3^ Department of Educational Media Development, Graduate School of Medical and Dental Sciences, Tokyo Medical and Dental University, Tokyo, Japan.; ^4^ Department of Behavioral Dentistry, Graduate School of Medical and Dental Sciences, Tokyo Medical and Dental University, Tokyo, Japan.

**Keywords:** regeneration, stem cells, tissue engineering

## Abstract

Periodontitis is a chronic inflammatory disease that induces the destruction of tooth-supporting tissues, followed by tooth loss. Although several approaches have been applied to periodontal regeneration, complete periodontal regeneration has not been accomplished. Tissue engineering using a combination of cells and scaffolds is considered to be a viable alternative strategy. We have shown that autologous transplantation of periodontal ligament-derived multipotent mesenchymal stromal cell (PDL-MSC) sheets regenerates periodontal tissue in canine models. However, the indications for autologous cell transplantation in clinical situations are limited. Therefore, this study evaluated the safety and efficacy of allogeneic transplantation of PDL-MSC sheets using a canine horizontal periodontal defect model. Canine PDL-MSCs were labeled with enhanced green fluorescent protein (EGFP) and were cultured on temperature-responsive dishes. Three-layered cell sheets were transplanted around denuded root surfaces either autologously or allogeneically. A mixture of β-tricalcium phosphate and collagen gel was placed on the bone defects. Eight weeks after transplantation, dogs were euthanized and subjected to microcomputed tomography and histological analyses. RNA and DNA were extracted from the paraffin sections to verify the presence of EGFP at the transplantation site. Inflammatory markers from peripheral blood sera were quantified using an enzyme-linked immunosorbent assay. Periodontal regeneration was observed in both the autologous and the allogeneic transplantation groups. The allogeneic transplantation group showed particularly significant regeneration of newly formed cementum, which is critical for the periodontal regeneration. Serum levels of inflammatory markers from peripheral blood sera showed little difference between the autologous and allogeneic groups. EGFP amplicons were detectable in the paraffin sections of the allogeneic group. These results suggest that allogeneic PDL-MSC sheets promoted periodontal tissue regeneration without side effects. Therefore, allogeneic transplantation of PDL-MSC sheets has a potential to become an alternative strategy for periodontal regeneration.

## Introduction

Periodontitis is a chronic inflammatory disease caused by oral bacteria that induce the destruction of tooth-supporting tissues and tooth loss.^1^ It is also associated with systemic diseases such as diabetes mellitus and cardiovascular disease.^2,3^ Although guided tissue regeneration (GTR) and enamel matrix derivatives have been applied,^4,5^ current regenerative therapies have had limited success in accomplishing effective periodontal regeneration.^6^ It is difficult to recover the correct structure and function of periodontal tissue using existing therapies because of the complex structure of periodontal tissue.^7^ Tissue engineering using a combination of stem cells and scaffold is therefore expected to achieve complete regeneration.^8^ Various types of multipotent mesenchymal stromal cells (MSC) have already been applied to periodontal regeneration in clinical trials. Bone marrow-derived MSCs,^9^ periodontal ligament-derived MSCs,^10^ and alveolar periosteal cells^11,12^ are able to promote periodontal regeneration.

In our laboratory, a periodontal ligament-derived multipotent MSC (PDL-MSC) sheet was investigated for periodontal regeneration.^13^ The cell sheet technique is an attractive tool that utilizes temperature-responsive culture dishes.^14^ Intact cells with an abundant extracellular matrix can be obtained as a sheet simply by reducing the temperature.^15^ Our previous studies have demonstrated that autologous transplantation of PDL-MSC sheets regenerated periodontal tissue in canine models^16,17^ and that PDL-MSCs are a more suitable cell source for periodontal regeneration than bone marrow-derived MSCs or alveolar periosteal cells.^17^

Based on these promising results, a clinical trial using autologous PDL-MSC sheets has been initiated.^8^ Briefly, PDL-MSCs were obtained from extracted teeth, and three-layered PDL-MSC sheets were transplanted onto the cleaned dental root. According to this protocol, tooth extraction is necessary to obtain autologous PDL-MSCs in each case.^18^ However, the indications for autologous PDL-MSC transplantation in clinical situations are limited, and allogeneic transplantation is expected to expand. One explanation for these limitations is that autologous transplantation requires extra teeth that contain healthy periodontal tissue and do not involve occlusion,^8^ and few periodontal patients have such extra teeth. However, the wisdom teeth of young people without periodontitis are extracted routinely. Therefore, PDL-MSCs are a readily available cell source, and a large number of PDL-MSCs can be expanded from a single tooth,^19^ which can be cryopreserved until transplantation.^20^ PDL-derived stem cells are considered to be MSCs, and allogeneic transplantation of MSCs has become a prevailing strategy.^21^ In addition, PDL-MSCs are known to have immunosuppressive effects.^22,23^ For these reasons, allogeneic PDL-MSC transplantation is considered to be an alternative solution to the limitations of autologous transplantation.

Thus, the purpose of this study was to evaluate the safety and efficacy of allogeneic transplantation of PDL-MSC sheets using a canine critical-size supra-alveolar periodontal defect model (horizontal periodontal defect model), which was established for evaluating the periodontal regenerative properties of biomaterials.^24,25^ In addition, the fate of transplanted allogeneic PDL-MSCs was assessed.

## Materials and Methods

### Animals

Eight healthy beagle dogs (∼10 kg, 1- to 2-year-old males) were used in this study. Dogs exhibited intact teeth with a healthy periodontal tissue. All of the experimental protocols were approved by the Animal Welfare Committee of Tokyo Women's Medical University.

### Cell culture

All procedures in dogs were performed with general and local anesthesia. Dogs were intramuscularly injected with 0.1 mg/kg medetomidine (Domitor; Nippon Zenyaku Kogyo, Fukushima, Japan) and 15 mg/kg ketamine (Ketalal; Daiichi Sankyo Propharma, Tokyo, Japan) for anesthetic premedication, and then subjected to an intravenous injection of 2.5 mg/kg propofol (Diprivan; AstraZeneca, Osaka, Japan). An endotracheal tube was inserted, and anesthesia was maintained with sevoflurane (Sevofrane; Abbott Japan, Tokyo, Japan). Dental calculus was removed with an ultrasonic scaler. Local anesthesia was performed with 2% lidocaine hydrochloride containing epinephrine at a concentration of 1:80,000 (Xylocaine Cartridge for Dental Use; Dentsply-Sankin, Tokyo, Japan). Four weeks before transplantation, the first and second mandibular premolars and the maxillary first, second, and third premolars were extracted.

Primary culture was performed as described previously.^17^ One day after seeding, floating cells were removed, and the medium was replaced with complete fresh medium. Passages were performed every 4–5 days.

### Flow cytometry

One million cells from passage 4 were suspended in 500 μL of Dulbecco's phosphate-buffered saline (D-PBS; Invitrogen, Carlsbad, CA) incubated with each specific antibody. To evaluate surface markers, fluorescein isothiocyanate (FITC)—or phycoerythrin (PE)-coupled antibodies against CD29 (Acris Antibodies, San Diego, CA), CD34 (R&D Systems, Minneapolis, MN), CD45, and CD90 (eBioscience, San Diego, CA) were used. As isotype controls, FITC-coupled nonspecific mouse IgG (R&D Systems), rat IgG (eBioscience), and PE-coupled nonspecific rat IgG2b kappa (eBioscience) were substituted for primary antibodies. After incubation for 30 min at 4°C in the dark, cells were washed with D-PBS and then resuspended in 500 μL of D-PBS. Cell fluorescence was determined using a flow cytometer (Gallios; Beckman Coulter, Brea, CA).

### Differentiation assay

One hundred cells at passage 3 were plated in 60-cm^2^ dishes and cultured in complete medium for 10 days, as described previously.^19^ For osteogenesis studies, the medium was switched to osteogenic differentiation medium consisting of complete medium supplemented with 82 μg/mL L-ascorbic acid phosphate, magnesium salt n-hydrate (AA; Wako, Tokyo, Japan), 10 mmol/L β-glycerophosphate (β-GP) (Sigma-Aldrich, St. Louis, MO), and 100 nmol/L dexamethasone (DEX) (DEXART; Fuji Pharma, Toyama, Japan) for an additional 10 days. Next, dishes were stained with 1% Alizarin Red S solution. For adipogenesis studies, the medium was switched to adipogenic differentiation medium, which consisted of complete medium supplemented with 100 nmol/L DEX, 0.5 mmol/L isobutyl-1-methyl xanthine (Sigma-Aldrich), and 50 mmol/L indomethacin (Wako), for an additional 21 days. Adipogenic cultures were stained with fresh Oil Red O solution.

### Enhanced green fluorescent protein labeling

The open reading frame of enhanced green fluorescent protein (EGFP) was generated from pEGFP-C1 (Clontech, Palo Alto, CA) by polymerase chain reaction (PCR) using the following primers: 5′-CACCATGGTGAGCAAGGGCGA, sense; 5′-TCTAGATCCGGTGGATCCCG, antisense. The resulting PCR products were subcloned into pENTR/D-TOPO (Invitrogen). The clone generated in the entry vector was sequenced to confirm its proper orientation and sequence and was later subcloned into the Gateway pLenti6.3/V5-DEST expression vector (Invitrogen) using the LR Clonase II enzyme reaction in accordance with the manufacturer's instructions. Following the production of virus in 293FT cells with ViraPower packaging mix (Invitrogen), the PDL-MSCs (passage 2) from individual dogs were transduced with pLenti6.3/EGFP-V5 (multiplicity of infection [MOI]: 0.1). EGFP-positive cells were sorted by fluorescence-activated cell scanning (FACS) using the MoFlo XDP cell sorter (Beckman Coulter). Selected cells were used for the following assays and transplantation.

### Colony-forming assay

One hundred cells at passage 3 were plated in a 60-cm^2^ dish, cultured in complete medium for 14 days, and stained with 2% crystal violet in methanol (Kanto Chemical, Tokyo, Japan) for 5 min, as described previously.^26^ Cells were washed twice with distilled water, and the number of colonies was counted. Colonies <2 mm in diameter or with faint staining were ignored.

### Alkaline phosphatase activity

Cells at passage 5 were plated in a 96-well plate at a density of 1 × 10^4^ cells/0.32-cm^2^ and cultured in complete medium for 48 h. Next, the medium was switched to complete medium with or without osteoinductive supplements, 82 μg/mL AA, 10 mmol/L β-GP, and 10 nmol/L DEX. After 5 additional days, cells were washed twice with sterile saline, and alkaline phosphatase (ALP) activity was evaluated by the LabAssay ALP™ kit (Wako). Absorbance at 405 nm was read with a plate reader (Molecular Devices, Sunnyvale, CA) using SoftMax Pro software.

### Preparation of cell sheets

Cell sheets were prepared as described previously. Briefly, canine PDL-MSCs at passage 5 were seeded in temperature-responsive culture dishes (35 mm in diameter) (UpCell; CellSeed, Inc., Tokyo, Japan) at a cell density of 4 × 10^4^ cells/9 cm^2^ and cultured in complete medium with osteoinductive supplements, 82 μg/mL AA, 10 mmol/L β-GP, and 10 nmol/l DEX, for 8–10 days. To harvest the cell sheet, culture temperature was reduced to room temperature before the medium was aspirated and washed with sterile saline, and a polyglycolic acid (PGA) sheet (Neoveil^®^, Felt-Sheet Type, 0.15 mm thickness; Gunze, Osaka, Japan) was placed on the surface of the cell sheet in the culture dish as a reinforcement carrier. Cell sheets attached to PGA sheets were harvested by peeling them from the dishes with forceps, and were then placed on the other cell sheet in the culture dishes. This procedure was repeated two more times until three-layered cell sheets were fabricated.

### Quality evaluation of the cell sheets

PDL-MSC sheets from each dog were evaluated before transplantation. PDL-MSC sheets were treated with collagenase for 15 min and trypsin-EDTA for 5 min in accordance with a previously reported method.^27^ To evaluate the number of cells and cell survival rate of each cell sheet, cell suspensions were prepared from individual cell sheets. The EGFP-positive ratio in the PDL-MSC suspension was also measured by FACS analysis.

### Transplantation

All surgical procedures were performed under general and local anesthesia, as in the case of tooth extraction. Four weeks after tooth extraction, bilateral critical-size supra-alveolar defects were created surgically on the mandibular third and fourth premolars in each animal as reported previously.^28^ Four dogs (Dog No. 1–4) were assigned to the autologous PDL-MSC sheet transplantation group (autologous group) and the control group. One-sided defects of the other four dogs (Dog No. 5–8) were assigned to the allogeneic PDL-MSC sheet transplantation group (allogeneic group). The remaining defects were used for another study. Buccal and lingual full-thickness flaps were raised following sulcular incisions from the canine tooth to the second molar. The crowns of the teeth were cut to ∼2 mm coronal to the cementum–enamel junction (CEJ), and the cut surfaces were smoothed. Exposed dental pulp tissues were capped with a temporary filling material (Cavit-G^®^; 3M ESPE, St. Paul, MN). The maxillary fourth premolars were also reduced in height, and the exposed dental pulp tissues were capped with the material (Cavit-G) to exclude the potential trauma of the occlusion. The mandibular first molars were reduced to the level of the alveolar crest. Alveolar bone was removed around the mandibular third and fourth premolar teeth using bone chisels and dental water-cooled rotating burs. The critical defect height from the CEJ to the reduced alveolar crest was set at 6 mm (Fig. 3A). The root cementum was removed with periodontal curettes, bone chisels, and dental water-cooled rotating burs.

Exposed root surface was treated for 2 min with EDTA (PrefGel^®^; Straumann, Basel, Switzerland). After washing with saline, three-layered PDL-MSC sheets supported by a PGA sheet were trimmed to the size of the root defect and applied to the exposed root surfaces in the experimental group, while only PGA carriers were applied in the control group (Fig. 3B, G). Constructs of porous β-tricalcium phosphate (β-TCP, Osferion^®^, G1; Olympus Terumo Biomaterials, Tokyo, Japan) mixed with 3% type I collagen (β-TCP/collagen at a 1:1 ratio by weight; Koken, Tokyo, Japan) were placed to cover the roots as a substitute for the removed alveolar bone (Fig. 3C, H). The β-TCP/collagen component was covered with an absorbable GTR membrane (Biomend^®^; Zimmer Dental, Carlsbad, CA), which was wrapped over the component. The membrane was fixed to the reduced alveolar bone with bone tacks (ACE trueTACK™; ACE Surgical Supply, Brockton, MA) (Fig. 3D, H). Releasing incisions were performed to generate a tension-free flap. Gingival flaps were advanced to allow flap margins that were adjusted to be ∼3 mm coronal to the collagen membranes and sutured (Fig. 3E). As postsurgical management, rectal administration of 50 mg diclofenac sodium (Novaltis, Tokyo, Japan) was performed for postsurgery pain control and fed a soft diet for 8 weeks and were administered antibiotics perorally (azithromycin; Pfizer, Tokyo, Japan) at a dose of 250 mg per day for 3 days and topically with 5% (W/V) chlorhexidine gluconate solution (Hibitane^®^; Dainippon Sumitomo Pharma, Osaka, Japan) twice weekly for 8 weeks. Sutures were removed at 2 weeks after surgery.

### Enzyme-linked immunosorbent assay

The peripheral blood samples were collected from eight dogs at three specific time points: before transplantation (0 weeks); at suture removal (2 weeks); and at euthanasia (8 weeks). Blood was allowed to clot for 2 h at room temperature before centrifugation twice for 10 min at 1580 *g*. Sera were stored at −80°C until assayed. Serum C-reactive protein (CRP), serum interleukin-10 (IL-10), serum interferon-γ (IFN-γ), and serum cluster of differentiation 30 (CD30) were determined using commercially available canine-specific enzyme-linked immunosorbent assay (ELISA) kits: CRP canine high-sensitivity ELISA Kit (Helica Biosystems, Santa Ana, CA); Quantikine canine IL-10; canine IFN-γ (R&D Systems); and canine CD30 ELISA Kit (Novateinbiosciences, Cambridge, MA) (*n* = 7 or 8 dogs). All sera samples were analyzed in duplicate in accordance with the manufacturer's instructions.

### Microcomputed tomography analysis and histological evaluation of periodontal regeneration

At 8 weeks after transplantation, all animals were, performed with general anesthesia, euthanized with intravenous potassium chloride solution (Terumo, Tokyo, Japan). Surgical sites were dissected and then fixed with 4% paraformaldehyde (Muto Pure Chemicals, Tokyo, Japan). Specimens were analyzed using a microcomputed tomography (μCT) system (SkyScan 1076; Skyscan, Kontich, Belgium). After removal of bone tacks, specimens were decalcified in Morse's solution^29,30^ for 4 months and embedded in paraffin. Sections (6 μm) were cut in a buccal–lingual plane, stained with Azan, and observed with a microscope (Eclipse E800; Nikon, Tokyo, Japan). Sections containing a central portion in the mesial–distal direction were selected and identified by μCT images. Histometric analyses were performed using Photoshop CS6 software (Adobe, San Jose, CA). Mean values of each histometric parameter were calculated for the buccal and the lingual sides. The following parameters were measured by two examiners who were blinded to the experimental conditions: (1) newly formed cementum ratio (%), which is the distance between the bottom of the defect and the coronal extension of a continuous layer of newly formed cementum or cementum-like deposit on the planed root by defect height; (2) periodontal score (PDL score), which was obtained by grading the periodontal ligament with the reported scoring system outlined by Wikesjo et al.^31^; (3) bone regeneration ratio (%), which is the distance between the bottom of the bone defect and the coronal extension of newly formed alveolar bone by defect height; and (4) ankylosis, which is the combined linear heights of the ankylotic union between the regenerated alveolar bone and the planed root (Fig. 2I).

### Isolation of RNA and DNA, and PCR

Paraffin-embedded sections of the allogeneic transplantation group and normal canine periodontal tissue (negative control) were used to isolate RNA and DNA. Total RNA was isolated using the PureLink™ FFPE Total RNA Isolation Kit (Invitrogen), and cDNA was generated from total RNA (250 ng) using the SuperScript^®^ VILO™ cDNA Synthesis Kit (Invitrogen). DNA extraction was performed with the QIAamp DNA FFPE Tissue Kit (Qiagen, Valencia, CA). PCR with Platinum PCR SuperMix (Invitrogen) was performed as follows: 30 sec denaturation at 94°C, followed by 45 cycles or 35 cycles (DNA) of denaturation at 94°C for 15 sec, primer annealing at 55°C for 15 sec, and elongation at 72°C for 1 min. The final elongation was conducted at 72°C for 1 min. PCR primers were designed based on the mRNA sequences available in the GenBank database. Primer pairs were as follows: EGFP (178 bp, sense: 5′-CGACAACCACTACCTGAGCA, antisense: 5′-GGTACCGTCGACTGCAGAAT). PCR products were purified by gel electrophoresis with E-Gel SizeSelect 2% agarose gels (Invitrogen). To verify the sequences of amplicons, direct sequencing was performed with two primers (5′-ACATGGTCCTGCTGGAGTTC, 5′-GGTACCGTCGACTGCAGAAT).

### Statistical analyses

All of the numerical results are expressed as mean ± standard deviation. Histometric analyses were performed by one-way repeated analysis of variance followed by Fisher's protected least significant difference *post hoc* test. Numerical data from ELISA analyses were analyzed using Student's *t-*test. A *p*-value <0.05 was considered to be statistically significant.

## Results

### Preparation and characterization of PDL-MSCs

**FIG. 1.**
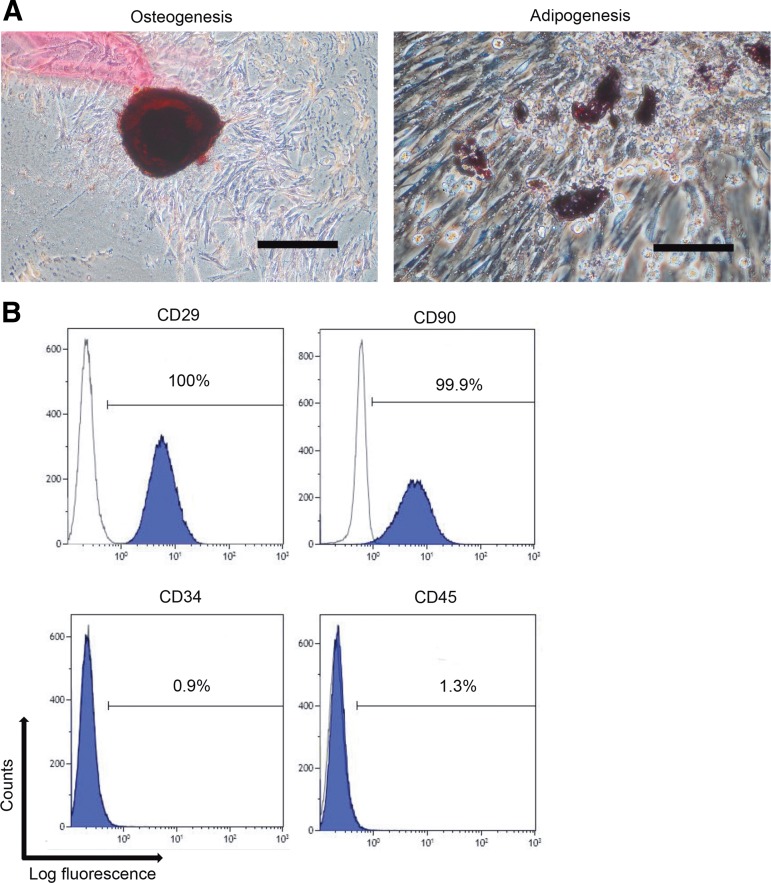
Dog PDL-MSCs possess multipotency. **(A)** The photograph on the left shows the results of the osteogenic differentiation assay using dog PDL-MSCs. PDL-MSCs were positively stained with Alizarin Red S. The photograph on the right shows the results of the adipogenic differentiation assay using dog PDL-MSCs. PDL-MSCs were positively stained with Oil Red O. Scale bars in the left and right photographs are 200 and 100 μm, respectively. **(B)** Surface molecule analysis by flow cytometry. PDL-MSCs were positive for CD29 and CD90 and negative for CD34 and CD45. PDL-MSC, periodontal ligament-derived multipotent mesenchymal stromal cell.

PDL-MSCs were collected from each dog and were successfully expanded *ex vivo*. PDL-MSCs showed osteogenic and adipogenic differentiation potential (Fig. 1A), were positive for the MSC markers, CD29 and CD90, and were negative for the hematopoietic stem cell markers, CD34 and CD45 (Fig. 1B).

**FIG. 2.**
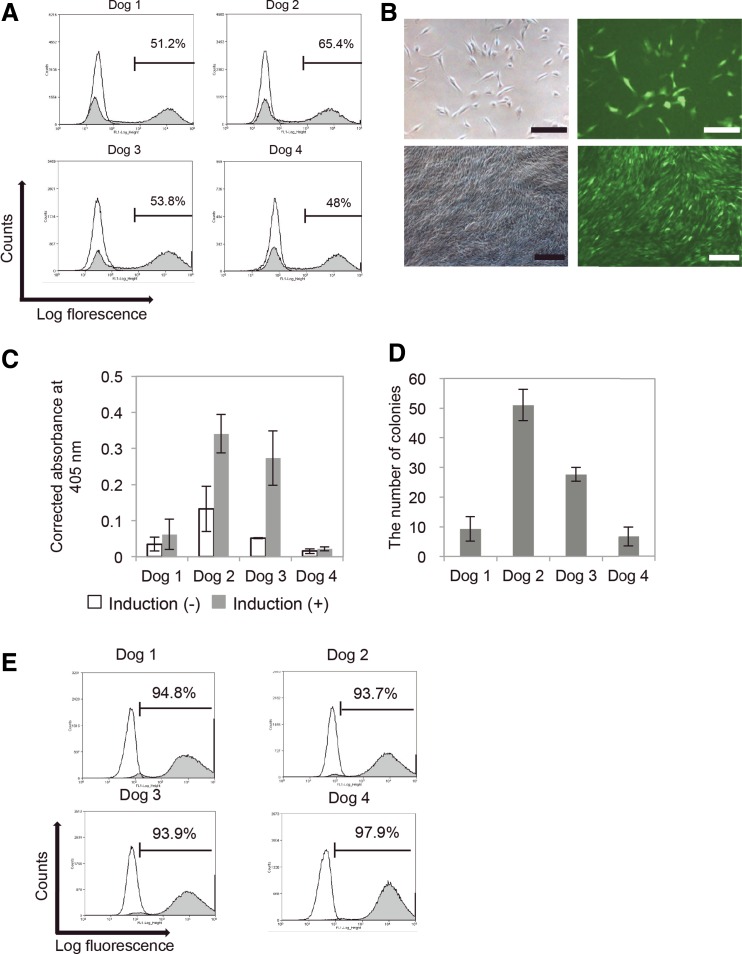
Preparation of dog PDL-MSC sheet with EGFP expression. **(A)** PDL-MSCs were labeled with EGFP by lentiviral transduction (the multiplicity of infection [MOI]: 0.1). Graphs show the results of FACS analyses of EGFP-positive PDL-MSCs from each dog. Only gated cells were sorted, and then used for the following assays and transplantation. **(B)** The photograph on the upper left shows PDL-MSCs that were labeled with EGFP by lentiviral transduction, and the photograph on the upper right shows the fluorescence of the same cells shown on the left. Scale bars in both photographs are 200 μm. The photograph on the lower left shows a PDL-MSC sheet that was labeled with EGFP by lentiviral transduction, and the photograph on the lower right shows the fluorescence of the same cell sheet shown on the left. Scale bars in both photographs are 400 μm. **(C)** ALP activity. **(D)** Colony-forming ability. PDL-MSCs obtained from Dog No. 2 had the highest ALP activity and colony-forming ability among the four dogs. **(E)** The graphs show the results of FACS analyses of PDL-MSC sheets prepared from PDL-MSCs from each dog. EGFP-positive rates of PDL-MSC sheets were >93.7% in dog PDL-MSCs. ALP, alkaline phosphatase; EGFP, enhanced green fluorescent protein; FACS, fluorescence-activated cell scanning.

To investigate the fate of transplanted PDL-MSCs, PDL-MSCs were labeled with EGFP by lentiviral transduction. Because high MOI reduced cell proliferation, the MOI was set at 0.1; thus, only EGFP-positive cells were sorted using a cell sorter (Fig. 2A, B). ALP activities and colony numbers of PDL-MSCs varied among the dogs. Among the four dogs, the highest PDL-MSC values were observed in Dog 2 (Fig. 2C, D).

### Preparation of PDL-MSC sheets

PDL-MSC sheets were harvested from each cell. The EGFP-positive ratio for PDL-MSCs in the cell sheet was >93.7% in all canine PDL-MSCs (Fig. 2E).

The cell survival rate of PDL-MSCs in the cell sheet was also highest in Dog 2 (Table 1), which indicates that the PDL-MSCs of Dog 2 were the most suitable cell source for allogeneic transplantation.

**Table 1.** **Cell Viability in Periodontal Ligament-Derived Multipotent Mesenchymal Stromal Cell Sheets Among Dogs**

	EGFP-labeled cell sheet		Control cell sheet	
Dog No.	No. of cells/sheet 10^5^	Survival rate (%)	No. of cells/sheet 10^5^	Survival rate (%)
1	3.8	81	5.6	89
2	5.0	88	9.5	93
3	4.2	84	5.3	90
4	1.2	73	2.5	86

EGFP-labeled cell sheet refers to a PDL-MSC sheet with EGFP expression by lentiviral transduction. Control cell sheet was generated with PDL-MSCs that were not transduced with lentiviral vector. The highest cell survival rate for PDL-MSCs was observed in both cell sheets of Dog 2.

EGFP, enhanced green fluorescent protein; PDL-MSC, periodontal ligament-derived multipotent mesenchymal stromal cell.

### Allogeneic PDL-MSC sheet transplantation to regenerate periodontal tissue in a canine horizontal defect model

**FIG. 3.**
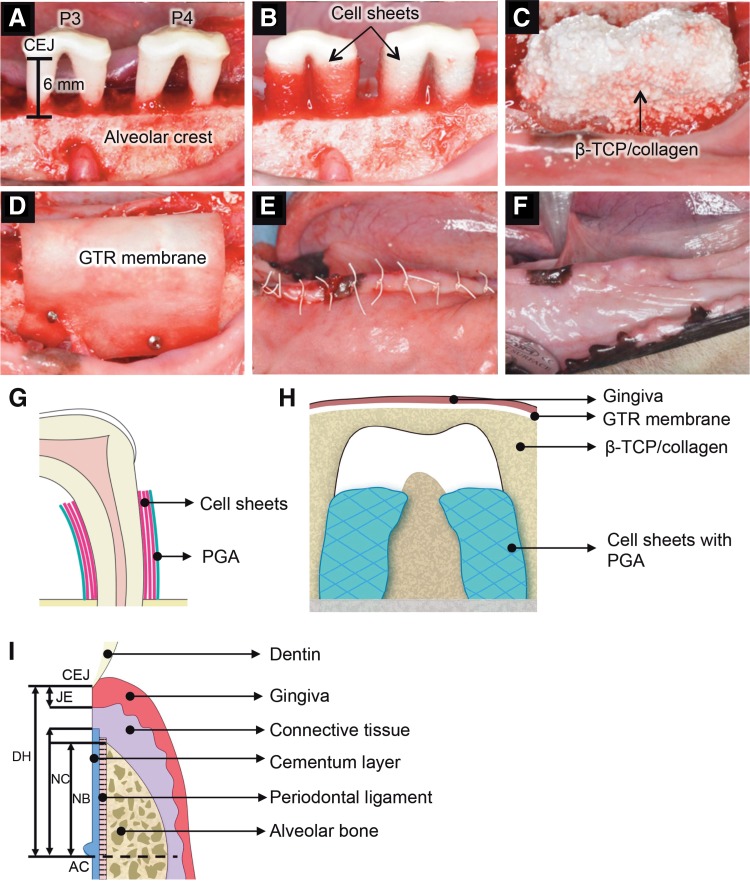
Allogeneic transplantation of PDL-MSC sheets in a canine horizontal defect model. The photographs **(A–E)** show the surgical procedures used for dogs. **(A)** Critical defect model. Alveolar bone was removed around the mandibular third and fourth premolars (P3 and P4). The defect height from the CEJ to the reduced alveolar crest was set at 6 mm. **(B)** PDL-MSC sheets were applied to the exposed root surfaces. **(C)** Constructs of β-TCP mixed with type I collagen were placed to cover the roots. **(D)** Absorbable guided tissue regeneration (GTR) membrane was wrapped over β-TCP. The membrane was fixed to the reduced alveolar bone with bone tacks. **(E)** The gingival flap was pulled to allow the flap margin to be ∼3 mm coronal to the collagen membranes and then sutured. **(F)** At 8 weeks after transplantation, no visible infection, suppuration, or adverse reactions were observed. **(G)** Schematic illustration of three-layered cell sheets and polyglycolic acid (PGA) sheet. **(H)** Schematic illustration of transplanted site. **(I)** The illustration shows the histometric parameters. B-TCP, β-tricalcium phosphate; CEJ, cementum–enamel junction; DH, defect height; JE, junctional epithelium; NC, newly formed cementum; NB, newly formed bone; AC, alveolar crest (bottom of bone defect).

After transplantation of PDL-MSC sheets, healing was found to progress uneventfully without an intense inflammatory reaction during an 8-week observation period. No visible infection, suppuration, or adverse reactions were observed for 8 weeks after surgery in either the autologous or allogeneic transplantation group (Fig. 3F). Dog 1 showed root exposure only in the control group.

**FIG. 4.**
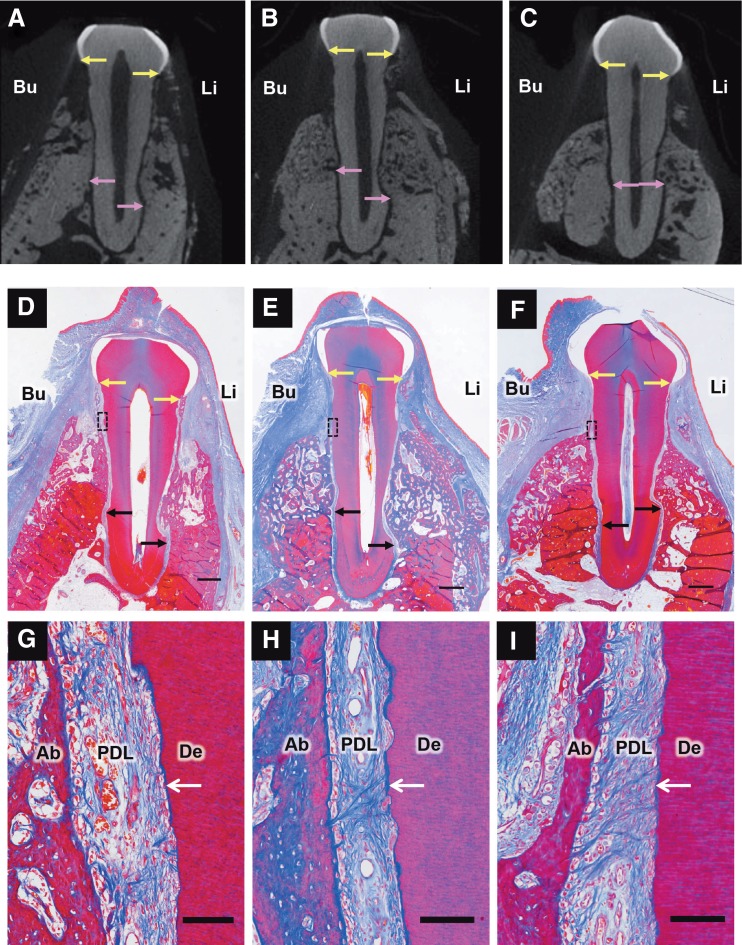
Images of μCT and histological specimens. The μCT images **(A–C)** show alveolar bone regeneration in the control, autologous, and allogeneic groups, respectively. Azan-stained tissue photographs in the middle **(D–F)** show alveolar bone regeneration in the control, autologous, and allogeneic groups, respectively. Black rectangles in photographs **(D–F)** are expanded in photographs **(G–I)**, respectively. **(G, H)** In the control and autologous groups, collagen fibers were aligned parallel or oblique to the denuded root surface. **(I)** In the allogeneic group, dense collagen fibers, which were attached perpendicularly to the cementum-like tissue layer, were observed. Scale bars in **(D–I)** are 1 mm and 100 μm, respectively. Yellow arrows in the photographs in the upper and middle rows show the CEJ. Pink and black arrows show the bottom of the bone defect. Bu and Li in the upper and middle rows indicate the buccal and lingual side, respectively. Ab, PDL, and De in the photographs in the lower row indicate alveolar bone, periodontal ligament, and dentin, respectively. White arrows in the photographs in the lower row indicate newly formed cementum. μCT, microcomputed tomography.

Alveolar bone regeneration was observed in all groups (Fig. 4A–F).

In the allogeneic group, dense collagen fibers were observed in the middle portion of the defect, which attached perpendicularly to the cementum-like tissue layer (Fig. 4H). In the autologous and control groups, collagen fibers were oriented obliquely or parallel to the root surface (Fig. 4G, I).

**FIG. 5.**
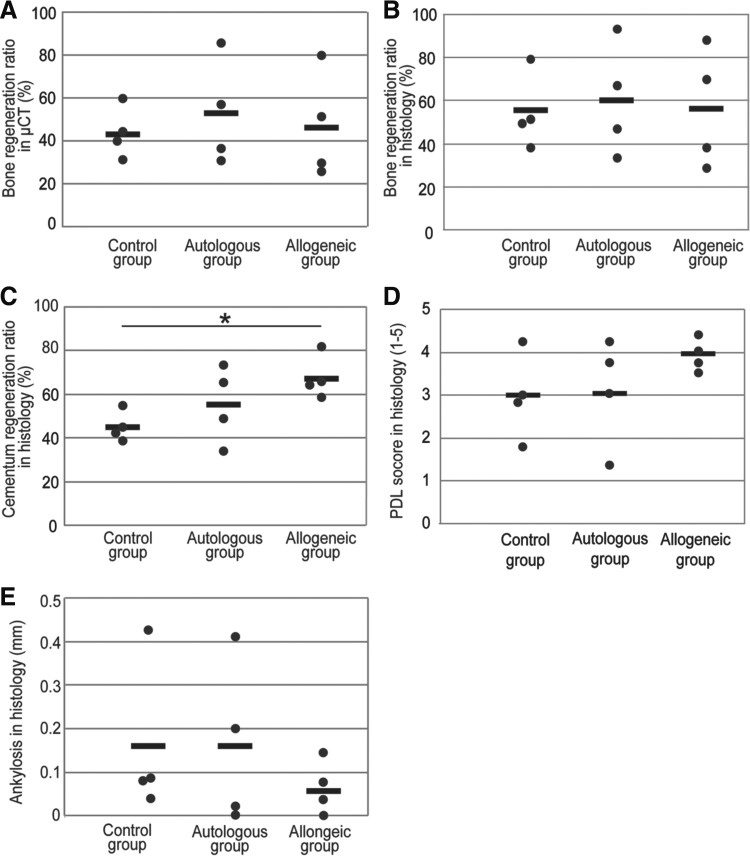
Numerical analyses of data obtained from the images of μCT and histological specimens of regenerated tissues. Graphs **(A, B)** show the bone regeneration ratios in μCT and histological analyses, respectively. There were no significant differences among the groups. Graph **(C)** shows the newly formed cementum regeneration ratio. Data values obtained for the allogeneic group were significantly higher than those of the control group. Graphs **(D, E)** show PDL score and ankylosis, respectively. There were no marked differences among the groups. Closed circles and thick black bars indicate actual values and averaged values, respectively. Asterisks (*) indicate *p* < 0.05.

The results of histometric analyses are shown in Figure 4. There were no significant differences between the autologous and allogeneic groups in the histometric analyses. No significant differences were observed with respect to the height of bone regeneration on CT and histometric analyses (Fig. 5A, B). The newly formed cementum regeneration ratio in the allogeneic group was significantly higher than that in the control group (*p* < 0.05) (Fig. 5C). PDL score and ankylosis showed no significant differences among the groups (Fig. 5D, E).

### Immunological reaction caused by autologous and allogeneic transplantation of PDL-MSC sheets

**FIG. 6.**
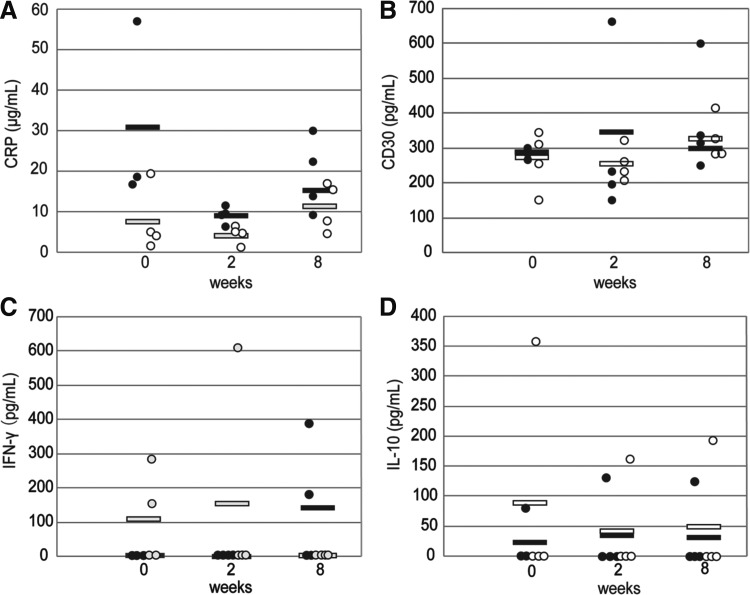
Evaluation of immunological reaction in response to allogeneic and autologous transplantation of periodontal ligament cell sheets by enzyme-linked immunosorbent assay. **(A)** Concentrations of serum C-reactive protein (mg/dL). **(B)** Concentrations of serum cluster of differentiation 30 (pg/mL). **(C)** Concentrations of serum interferon-γ (pg/mL). **(D)** Concentrations of serum interleukin-10 (pg/mL). **(A–D)** There were no significant differences in the concentrations of these inflammatory markers between the allogeneic and autologous groups at any time point. Open and closed circles indicate actual values for the allogeneic and autologous groups, respectively. Open and closed bars indicate averaged values for the allogeneic and autologous groups, respectively.

To confirm the potential immunological reactions due to transplantation of allogeneic PDL-MSC sheets, the presence of inflammatory signs was evaluated by ELISA. There were no significant differences in the concentrations of serum CRP, IFN-γ, IL-10, or CD30 between the allogeneic and autologous groups (*p* > 0.05) at each time point (Fig. 6A–D).

### Allogeneic transplanted PDL-MSCs remained in regenerated tissue

**FIG. 7.**
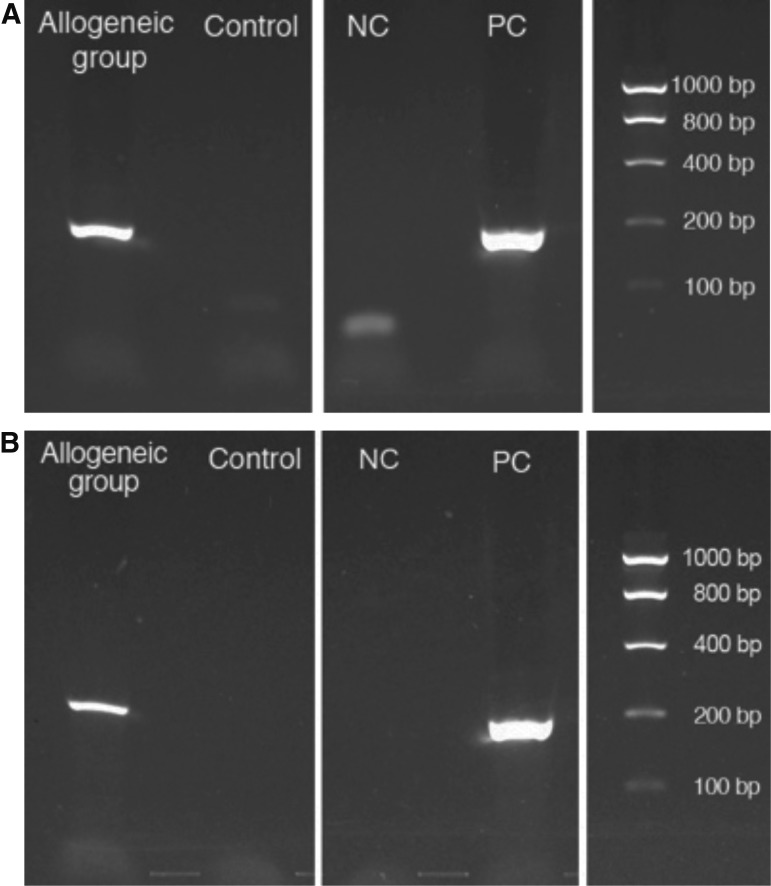
EGFP-positive signals in regenerated periodontal tissue. **(A)** The EGFP amplicon was detected at the RNA level in tissue that was transplanted allogeneically. **(B)** The EGFP amplicon was detected at the DNA level in tissue that was transplanted allogeneically. NC and PC indicate the negative control and positive control, respectively. Negative control was distilled water that was alternatively used for RNA and DNA. Positive control was RNA and DNA from EGFP-transduced dog periodontal ligament cells.

To investigate the fate of allogeneic transplanted PDL-MSCs, EGFP amplicons in the regenerated periodontal tissue were analyzed. PCR revealed that the mRNA and genomic DNA of EGFP resided in the tissue of the allogeneic group (Fig. 7A, B). Sequences of the PCR products were homologous with those of EGFP (data not shown).

## Discussion

Some reports have demonstrated that allogeneic transplantation of PDL-MSCs is useful for periodontal regeneration in a miniature pig model.^23,32^ However, these studies used two-wall defects, and the properties of the allogeneic cell sources were not confirmed. Therefore, in the present study, a critical periodontal defect model was used and the properties of allogeneic transplanted PDL-MSCs were examined. First, we confirmed that the harvested cells were MSCs outlined by the Mesenchymal and Tissue Stem Cell Committee of the International Society for Cellular Therapy.^33^

We transplanted autologous and allogeneic PDL-MSC sheets into each dog. On CT and histological analyses, all groups showed high bone regeneration ratios. This observation might be explained by the combined use of β-TCP/collagen and is consistent with a previous study.^17^

The allogeneic group exhibited higher cementum regeneration than the control group. Cementum regeneration is a vital step in periodontal regeneration.^34,35^ Previous research has shown that transplantation of PDL cell sheets accelerates cementum regeneration.^17,36,37^ Other parameters in the allogeneic and autologous groups also tended to be superior to those of the control group, as reported previously.^16,17^ However, no significant differences were detected. One explanation for this result may be the reduction in cell viability due to lentiviral transduction. Some reports have shown that lentiviral transduction causes a decrease in cell viability and cell proliferation.^38,39^ We also confirmed that the cell viability of EGFP-labeled cell sheets was lower compared with the control that was not transduced with lentiviral vector (Table 1).

A second explanation is the difference in the defect size when compared with previous studies. A critical defect is too large to provide adequate nutrients and blood supply, and it is therefore possible that the cells, particularly in the coronal region, would not survive under such conditions.

Some results also indicated that the allogeneic group tended to be superior to the autologous group, which might be due to the properties of the cell sheet. In this case, PDL-MSCs expressing a large number of colony-forming assays and elevated ALP activity were selected for allogeneic transplantation. Supplying cells with a uniform quality is considered to be an important factor in the success of allogeneic transplantation.^40,41^ Therefore, selecting cells with high quality for transplantation might provide effective results in the allogeneic group. Recently, the criteria for the human bone marrow MSCs have been reported.^42^ The research showed that the potency assay using the criteria is needed for the success of the regenerative medicine. Therefore, further research is needed to define the criteria of the PDL-MSCs.

After transplantation, none of the dogs showed adverse effects on a macroscopic level. Immunological reactions were investigated by ELISA. Peripheral blood serum levels of CRP, CD30, IL-10, and IFN-γ were examined, and there were no significant differences between the allogeneic and autologous groups. These results indicate that allogeneic transplanted cells did not cause immune rejection. No immunosuppressive treatment was performed in this study. Therefore, the lack of any immunoreaction might be due to the immunosuppressive effects of PDL-MSCs. The immunomodulatory properties of MSCs have been reported,^43–45^ while PDL-MSCs have also been reported to have immunosuppressive activity *in vitro.*^22,23,32^ The immunosuppressive function of PDL-MSCs may have affected the present results similarly to previously reports.^22,32^ In addition, it was recently reported that MSCs have inflammatory-modulating effects.^46^ This may also have contributed to the low elevation of inflammatory markers. Another reason for the lack of immunoreaction may be the use of a closed beagle dog colony. However, there have been some reports that the major histocompatibility complex (MHC) diversity of dogs is not necessarily related to immune competence^47^ or the results of allogeneic transplantation.^48,49^ Further investigation is necessary to reveal the involvement of dog MHC diversity.

In the present study, we also investigated the fate of transplanted PDL-MSCs by lentiviral transduction of the EGFP gene. Previous studies have reported that transplanted PDL-MSCs differentiate into periodontal tissue in a porcine model^23^ or rat model.^50^ However, other studies have shown that transplanted cells are lost over the short term, and paracrine effects are important in MSC transplantation.^51^ Nevertheless, no studies have investigated the fate of allogeneic PDL-MSCs in a dog horizontal model. Although we were unable to detect EGFP protein by immunohistochemistry, long-term decalcification by strong acid may have caused the disappearance of EGFP expression at the protein level because several articles indicate that the decalcification by strong acid reduces the protein or immunoreactivity of tissue sections.^52,53^ However, decalcification and embedding paraffin of tissue are necessary to observe the clear soft and hard tissue surrounding teeth.^54^

EGFP amplicons were found in RNA or DNA samples, which were extracted from the histological samples of the allogeneic group. In Figure 7A, under negative control, there is a faint band that may be caused by the contamination of something during the making of cDNA from distilled water or nonpurified primer. In addition, PCR products were sequenced, and the results were homologous to the sequence of EGFP. These results indicate that the transplanted allogeneic PDL-MSCs remained alive or at least exhibited transcription at 8 weeks after cell transplantation.

## Conclusion

Allogeneic transplantation of PDL-MSC sheets is safe and effective for periodontal regeneration in a canine critical-size supra-alveolar periodontal defect model (horizontal periodontal defect model). Allogeneic PDL-MSCs are considered to be effective for direct periodontal regeneration.

## Abbreviations Used

AA: L-ascorbic acid phosphate, magnesium salt n-hydrate
ALP: alkaline phosphatase
CD30: cluster of differentiation 30
CEJ: cementum–enamel junction
CRP: C-reactive protein
DEX: dexamethasone
D-PBS: Dulbecco's phosphate-buffered saline
EGFP: enhanced green fluorescent protein
ELISA: enzyme-linked immunosorbent assay
FACS: fluorescence-activated cell scanning
FITC: fluorescein isothiocyanate
β-GP: β-glycerophosphate
GTR: guided tissue regeneration
IFN-γ: interferon-γ
IL-10: interleukin-10
μCT: microcomputed tomography
MHC: major histocompatibility complex
MOI: multiplicity of infection
MSC: mesenchymal stromal cell
PCR: polymerase chain reaction
PDL-MSC: periodontal ligament-derived multipotent mesenchymal stromal cell
PE: phycoerythrin
PGA: polyglycolic acid
β-TCP: β-tricalcium phosphate

## References

[1] Hajishengallis G, Darveau RP, Curtis MA The keystone-pathogen hypothesis. Nat Rev Microbiol. 2012;10:717–725 22941505 10.1038/nrmicro2873 PMC3498498

[2] Lalla E, Papapanou PN Diabetes mellitus and periodontitis: a tale of two common interrelated diseases. Nat Rev Endocrinol. 2011;7:738–748 21709707 10.1038/nrendo.2011.106

[3] Lockhart PB, Bolger AF, Papapanou PN, et al. Periodontal disease and atherosclerotic vascular disease: does the evidence support an independent association?: a scientific statement from the American Heart Association. Circulation. 2012;125:2520–2544 22514251 10.1161/CIR.0b013e31825719f3

[4] Koop R, Merheb J, Quirynen M Periodontal regeneration with enamel matrix derivative in reconstructive periodontal therapy: a systematic review. J Periodontol. 2012;83:707–720 22050544 10.1902/jop.2011.110266

[5] Ramseier CA, Rasperini G, Batia S, et al. Advanced reconstructive technologies for periodontal tissue repair. Periodontol 2000. 2012;59:185–202 22507066 10.1111/j.1600-0757.2011.00432.x PMC3335769

[6] Chen FM, Sun HH, Lu H, et al. Stem cell-delivery therapeutics for periodontal tissue regeneration. Biomaterials. 2012;33:6320–6344 22695066 10.1016/j.biomaterials.2012.05.048

[7] Chen FM, Jin Y Periodontal tissue engineering and regeneration: current approaches and expanding opportunities. Tissue Eng Part B Rev. 2010;16:219–255 19860551 10.1089/ten.TEB.2009.0562

[8] Iwata T, Washio K, Yoshida T, et al. Cell sheet engineering and its application for periodontal regeneration. J Tissue Eng Regen Med. 2015;9:343–356 23881816 10.1002/term.1785

[9] Yamada Y, Ueda M, Hibi H, et al. A novel approach to periodontal tissue regeneration with mesenchymal stem cells and platelet-rich plasma using tissue engineering technology: a clinical case report. Int J Periodontics Restorative Dent. 2006;26:363–369 16939018

[10] Feng F, Akiyama K, Liu Y, et al. Utility of PDL progenitors for in vivo tissue regeneration: a report of 3 cases. Oral Dis. 2010;16:20–28 20355278 10.1111/j.1601-0825.2009.01593.x PMC2848819

[11] Yamamiya K, Okuda K, Kawase T, et al. Tissue-engineered cultured periosteum used with platelet-rich plasma and hydroxyapatite in treating human osseous defects. J Periodontol. 2008;79:811–818 18454659 10.1902/jop.2008.070518

[12] Okuda K, Kawase T, Nagata M, et al. Tissue-engineered cultured periosteum sheet application to treat infrabony defects: case series and 5-year results. Int J Periodontics Restorative Dent. 2013;33:281–287 23593621 10.11607/prd.1545

[13] Ishikawa I, Iwata T, Washio K, et al. Cell sheet engineering and other novel cell-based approaches to periodontal regeneration. Periodontol 2000. 2009;51:220–238 19878477 10.1111/j.1600-0757.2009.00312.x

[14] Okano T, Yamada N, Sakai H, et al. A novel recovery system for cultured cells using plasma-treated polystyrene dishes grafted with poly(N-isopropylacrylamide). J Biomed Mater Res. 1993;27:1243–1251 8245039 10.1002/jbm.820271005

[15] Yang J, Yamato M, Shimizu T, et al. Reconstruction of functional tissues with cell sheet engineering. Biomaterials. 2007;28:5033–5043 17761277 10.1016/j.biomaterials.2007.07.052

[16] Iwata T, Yamato M, Tsuchioka H, et al. Periodontal regeneration with multi-layered periodontal ligament-derived cell sheets in a canine model. Biomaterials. 2009;30:2716–2723 19201461 10.1016/j.biomaterials.2009.01.032

[17] Tsumanuma Y, Iwata T, Washio K, et al. Comparison of different tissue-derived stem cell sheets for periodontal regeneration in a canine 1-wall defect model. Biomaterials. 2011;32:5819–5825 21605900 10.1016/j.biomaterials.2011.04.071

[18] Washio K, Iwata T, Mizutani M, et al. Assessment of cell sheets derived from human periodontal ligament cells: a pre-clinical study. Cell Tissue Res. 2010;341:397–404 20632035 10.1007/s00441-010-1009-1

[19] Iwata T, Yamato M, Zhang Z, et al. Validation of human periodontal ligament-derived cells as a reliable source for cytotherapeutic use. J Clin Periodontol. 2010;37:1088–1099 20618549 10.1111/j.1600-051X.2010.01597.x

[20] Vasconcelos RG, Ribeiro RA, Vasconcelos MG, et al. In vitro comparative analysis of cryopreservation of undifferentiated mesenchymal cells derived from human periodontal ligament. Cell Tissue Bank. 2012;13:461–469 21833489 10.1007/s10561-011-9271-3

[21] Ankrum JA, Ong JF, Karp JM Mesenchymal stem cells: immune evasive, not immune privileged. Nat Biotechnol. 2014;32:252–260 24561556 10.1038/nbt.2816 PMC4320647

[22] Wada N, Menicanin D, Shi S, et al. Immunomodulatory properties of human periodontal ligament stem cells. J Cell Physiol. 2009;219:667–676 19160415 10.1002/jcp.21710

[23] Ding G, Liu Y, Wang W, et al. Allogeneic periodontal ligament stem cell therapy for periodontitis in swine. Stem Cells. 2010;28:1829–1838 20979138 10.1002/stem.512 PMC2996858

[24] Wikesjo UM, Guglielmoni P, Promsudthi A, et al. Periodontal repair in dogs: effect of rhBMP-2 concentration on regeneration of alveolar bone and periodontal attachment. J Clin Periodontol. 1999;26:392–400 10382580 10.1034/j.1600-051x.1999.260610.x

[25] Pellegrini G, Seol YJ, Gruber R, et al. Pre-clinical models for oral and periodontal reconstructive therapies. J Dent Res. 2009;88:1065–1076 19887682 10.1177/0022034509349748 PMC3318031

[26] Sakaguchi Y, Sekiya I, Yagishita K, et al. Comparison of human stem cells derived from various mesenchymal tissues: superiority of synovium as a cell source. Arthritis Rheum. 2005;52:2521–2529 16052568 10.1002/art.21212

[27] Washio K, Kuroda H, Iwata T, et al. Imporved enzymatic treatment for accurrate cell counting from extracellar matrix-rich periodontal ligament cell sheets. Int J Oral Maxillofac Implants. 2014;29:e117–e121 24451879 10.11607/jomi.te50

[28] Kwon DH, Bisch FC, Herold RW, et al. Periodontal wound healing/regeneration following the application of rhGDF-5 in a beta-TCP/PLGA carrier in critical-size supra-alveolar periodontal defects in dogs. J Clin Periodontol. 2010;37:667–674 20492073 10.1111/j.1600-051X.2010.01569.x

[29] Morse A Formic acid–sodium citrate decalcification and butyl alcohol dehydration of teeth and bones for sectioning in paraffin. J Dent Res. 1945;24:143–153

[30] Nakatomi M, Morita I, Eto K, et al. Sonic hedgehog signaling is important in tooth root development. J Dent Res. 2006;85:427–431 16632755 10.1177/154405910608500506

[31] Wikesjo UM, Sorensen RG, Kinoshita A, et al. Periodontal repair in dogs: effect of recombinant human bone morphogenetic protein-12 (rhBMP-12) on regeneration of alveolar bone and periodontal attachment. J Clin Periodontol. 2004;31:662–670 15257745 10.1111/j.1600-051X.2004.00541.x

[32] Liu O, Xu J, Ding G, et al. Periodontal ligament stem cells regulate B lymphocyte function via programmed cell death protein 1. Stem Cells. 2013;31:1371–1382 23553748 10.1002/stem.1387

[33] Dominici M, Le Blanc K, Mueller I, et al. Minimal criteria for defining multipotent mesenchymal stromal cells. The International Society for Cellular Therapy position statement. Cytotherapy. 2006;8:315–317 16923606 10.1080/14653240600855905

[34] Bartold PM, McCulloch CA, Narayanan AS, et al. Tissue engineering: a new paradigm for periodontal regeneration based on molecular and cell biology. Periodontol 2000. 2000;24:253–269 11276871 10.1034/j.1600-0757.2000.2240113.x

[35] Polimeni G, Xiropaidis AV, Wikesjo UM Biology and principles of periodontal wound healing/regeneration. Periodontol 2000. 2006;41:30–47 16686925 10.1111/j.1600-0757.2006.00157.x

[36] Akizuki T, Oda S, Komaki M, et al. Application of periodontal ligament cell sheet for periodontal regeneration: a pilot study in beagle dogs. J Periodontal Res. 2005;40:245–251 15853971 10.1111/j.1600-0765.2005.00799.x

[37] Flores MG, Yashiro R, Washio K, et al. Periodontal ligament cell sheet promotes periodontal regeneration in athymic rats. J Clin Periodontol. 2008;35:1066–1072 19040584 10.1111/j.1600-051X.2008.01326.x

[38] Lotharius J, Barg S, Wiekop P, et al. Effect of mutant alpha-synuclein on dopamine homeostasis in a new human mesencephalic cell line. J Biol Chem. 2002;277:38884–38894 12145295 10.1074/jbc.M205518200

[39] Godlewski J, Nowicki MO, Bronisz A, et al. Targeting of the Bmi-1 oncogene/stem cell renewal factor by microRNA-128 inhibits glioma proliferation and self-renewal. Cancer Res. 2008;68:9125–9130 19010882 10.1158/0008-5472.CAN-08-2629

[40] Imanishi Y, Saito A, Komoda H, et al. Allogenic mesenchymal stem cell transplantation has a therapeutic effect in acute myocardial infarction in rats. J Mol Cell Cardiol. 2008;44:662–671 18343403 10.1016/j.yjmcc.2007.11.001

[41] Atoui R, Chiu RC Concise review: immunomodulatory properties of mesenchymal stem cells in cellular transplantation: update, controversies, and unknowns. Stem Cells Transl Med. 2012;1:200–205 23197779 10.5966/sctm.2011-0012 PMC3659851

[42] Samsonraj RM, Rai B, Sathiyanathan P, et al. Establishing criteria for human mesenchymal stem cell potency. Stem Cells. 2015;33:1878–1891 25752682 10.1002/stem.1982 PMC5363381

[43] Aggarwal S, Pittenger MF Human mesenchymal stem cells modulate allogeneic immune cell responses. Blood. 2005;105:1815–1822 15494428 10.1182/blood-2004-04-1559

[44] English K, French A, Wood KJ Mesenchymal stromal cells: facilitators of successful transplantation? Cell Stem Cell. 2010;7:431–442 20887949 10.1016/j.stem.2010.09.009

[45] English K, Mahon BP Allogeneic mesenchymal stem cells: agents of immune modulation. J Cell Biochem. 2011;112:1963–1968 21445861 10.1002/jcb.23119

[46] Lee RH, Yu JM, Foskett AM, et al. TSG-6 as a biomarker to predict efficacy of human mesenchymal stem/progenitor cells (hMSCs) in modulating sterile inflammation in vivo. Proc Natl Acad Sci U S A. 2014;111:16766–16771 25385603 10.1073/pnas.1416121111 PMC4250139

[47] Wade CM Inbreeding and genetic diversity in dogs: results from DNA analysis. Vet J. 2011;189:183–188 21745753 10.1016/j.tvjl.2011.06.017

[48] Tillson M, Niemeyer GP, Welch JA, et al. Hematopoietic chimerism induces renal and skin allograft tolerance in DLA-identical dogs. Exp Hematol. 2006;34:1759–1770 17157174 10.1016/j.exphem.2006.08.004

[49] Liu G, Zhang Y, Liu B, et al. Bone regeneration in a canine cranial model using allogeneic adipose derived stem cells and coral scaffold. Biomaterials. 2013;34:2655–2664 23343633 10.1016/j.biomaterials.2013.01.004

[50] Han J, Menicanin D, Marino V, et al. Assessment of the regenerative potential of allogeneic periodontal ligament stem cells in a rodent periodontal defect model. J Periodontal Res. 2014;49:333–345 23841948 10.1111/jre.12111

[51] Caplan AI, Correa D The MSC: an injury drugstore. Cell Stem Cell. 2011;9:11–15 21726829 10.1016/j.stem.2011.06.008 PMC3144500

[52] Athanasou NA, Quinn J, Heryet A, et al. Effect of decalcification agents on immunoreactivity of cellular antigens. J Clin Pathol. 1987;40:874–878 2443541 10.1136/jcp.40.8.874 PMC1141128

[53] Hosoya A, Hoshi K, Sahara N, et al. Effects of fixation and decalcification on the immunohistochemical localization of bone matrix proteins in fresh-frozen bone sections. Histochem Cell Biol. 2005;123:639–646 15940506 10.1007/s00418-005-0791-4

[54] Keklikoglu N, Akinci S Comparison of three different techniques for histological tooth preparation. Folia Histochem Cytobiol. 2013;51:286–291 24497133 10.5603/FHC.2013.0039

